# Supplementary material for: The influence of stand composition and season on canopy structure and understory light environment in different subtropical montane Pinus massoniana forests
Source: PeerJ. 2024 Mar 15;12:e17067. doi: 10.7717/peerj.17067 (PMC10946397; doi:10.7717/peerj.17067)
Supplement: Supplemental Information 1 [file peerj-12-17067-s001.docx]

| **Forest stand** | **Season** | **Index** | **Median** | **Mean** | **Min** | **Max** | **SD** | **CV (%)** |
| --- | --- | --- | --- | --- | --- | --- | --- | --- |
| PF | Summer | CO | 29.32 | 28.70 | 20.90 | 33.85 | 2.67 | 9.3 |
|  |  | LAI | 1.38 | 1.41 | 1.06 | 2.21 | 0.24 | 16.7 |
|  |  | Tdir | 7.46 | 7.37 | 2.03 | 11.76 | 1.66 | 22.5 |
|  |  | Tdif | 7.01 | 6.78 | 4.03 | 8.19 | 0.92 | 13.6 |
|  |  | Ttot | 14.34 | 14.15 | 6.06 | 19.66 | 2.31 | 16.3 |
|  | Winter | CO | 31.02 | 30.53 | 19.88 | 35.71 | 2.71 | 8.9 |
|  |  | LAI | 1.26 | 1.32 | 0.98 | 2.33 | 0.22 | 17.1 |
|  |  | Tdir | 5.50 | 5.62 | 2.54 | 9.66 | 1.50 | 26.6 |
|  |  | Tdif | 5.64 | 5.53 | 2.75 | 7.21 | 0.71 | 12.8 |
|  |  | Ttot | 11.18 | 11.16 | 6.69 | 16.83 | 1.90 | 17.0 |
| MF | Summer | CO | 22.44 | 22.87 | 15.94 | 35.15 | 3.21 | 14.0 |
|  |  | LAI | 1.69 | 1.71 | 1.11 | 2.54 | 0.28 | 16.1 |
|  |  | Tdir | 5.50 | 5.58 | 2.35 | 11.23 | 1.84 | 32.9 |
|  |  | Tdif | 5.22 | 5.29 | 3.19 | 8.13 | 0.98 | 18.5 |
|  |  | Ttot | 10.67 | 10.87 | 6.17 | 18.44 | 2.59 | 23.9 |
|  | Winter | CO | 26.72 | 26.70 | 17.89 | 34.24 | 3.25 | 12.2 |
|  |  | LAI | 1.54 | 1.55 | 1.07 | 2.34 | 0.28 | 17.8 |
|  |  | Tdir | 4.43 | 4.54 | 1.84 | 9.20 | 1.54 | 33.9 |
|  |  | Tdif | 4.66 | 4.71 | 2.61 | 6.78 | 0.90 | 19.0 |
|  |  | Ttot | 8.99 | 9.25 | 5.70 | 15.57 | 2.02 | 21.8 |
